# Rare giant ovarian metastasis arising from a small primary lung adenocarcinoma: a case report

**DOI:** 10.3389/fsurg.2023.1278076

**Published:** 2023-09-12

**Authors:** Baofeng Wang, Youjuan Jia, Jiang Wang, Zhenjiang Zhang, Yilin Ding, Hengxiao Lu

**Affiliations:** ^1^ Department of Thoracic Surgery, The First Affiliated Hospital of Weifang Medical University (Weifang people’s Hospital), School of Clinical Medicine, Weifang Medical University, Weifang, China; ^2^ Department of Gynecology, The First Affiliated Hospital of Weifang Medical University (Weifang people’s Hospital), Weifang, China; ^3^ Department of Thoracic Surgery, The First Affiliated Hospital of Weifang Medical University (Weifang people’s Hospital), Weifang, China

**Keywords:** lung adenocarcinoma (LUAD), ovary metastasis, anaplastic lymphoma kinase gene (ALK), immunohistochemical staining, tumor markers

## Abstract

This intricate case report details an exceptionally rare incidence of ovarian metastasis originating from a primary lung adenocarcinoma (LUAD). The relative rarity of this metastatic pathway in medical literature indicates significant diagnostic challenges. This patient was initially found to have both the ovarian tumor and lung nodule and they were originally considered independent primary tumors, derived from radiological interpretations and biomarker profiling. Nevertheless, subsequent postoperative histopathological and immunohistochemical staining evaluations identified ovarian tumors as invasive adenocarcinoma metastasized from lung. The lung and ovary tumor both showed marked anaplastic lymphoma kinase gene (ALK) protein expression by immunohistochemistry. The molecular pathologic genetic testing for lung tumor also revealed ALK rearrangement positive. The complexity of this case underscores the essentiality of maintaining a high degree of diagnostic vigilance, particularly when confronting synchronous tumors. In addition, immunohistochemical staining plays an important role in diagnosing the ovarian neoplasm's metastatic nature and determining the primary site of metastatic adenocarcinoma. For lung cancer with ovary metastasis patients, the adopting an adaptable treatment approach responsive to evolving diagnostic evidence can improve the accuracy of diagnosis and avoid excessive treatment of patients.

## Introduction

Lung carcinoma frequently manifests metastatic characteristics, commonly involving osseous structures, pulmonary tissues, cerebral matter, adrenal glands, hepatic tissue, and extrathoracic lymph nodes (1). Existing literature has documented instances of lung adenocarcinoma metastasizing to diverse sites including the colon, mammary tissue, pituitary gland, and gingival structures (2, 3). Nonetheless, the phenomenon of ovarian metastasis originating from lung adenocarcinoma presents as an exceptionally infrequent event of notable clinical intrigue. The scarceness of this event necessitates a comprehensive understanding of its clinical manifestations and treatment paradigms to forestall potential misdiagnosis in clinical practice.

In the presence of synchronous tumors, imaging techniques (ultrasound, computed tomography, positron emission tomography-computed tomography), and even conventional morphology are often inadequate for reliable diagnosis. In these circumstances, the use of appropriate immunohistochemical markers is able to provide additional evidence to differentiate primary from metastatic neoplasms.

In the case report herein, the patient initially manifested with a considerable ovarian neoplasm coupled with a pulmonary nodule. The prevailing hypothesis, grounded in contemporary medical literature, suggested these two pathologies as distinct entities, devoid of any metastatic interconnection. In the subsequent clinical course, the patient underwent an ovarian surgical procedure. The ensuing histopathological examination revealed an unexpected finding: the ovarian neoplasm had originated from pulmonary tissue, indicating a previously unidentified metastatic pathway. This newfound understanding led to a reevaluation of the patient's condition, subsequently necessitating thoracic surgery to address the primary lung malignancy. This intricate medical trajectory highlights the importance of maintaining diagnostic vigilance and the potential for clinical paradigms to evolve in the face of novel pathological evidence.

## Case presentation

A 43-year-old female presented to our institute with a primary complaint of unrelenting hypogastric pain for a duration of 11 days. Physical examination revealed normal vulvar development, vaginal patency, and ectropion of cervical columnar epithelium. The uterus was noted to be anteverted with a typical shape and size, possessing unrestricted mobility and no associated tenderness. A significant finding was the palpation of a hard, poorly mobile, and tender mass, approximately 10 cm in diameter, in the right adnexal area. Conversely, the left adnexal region demonstrated no palpable abnormalities.

**Figure 1:**
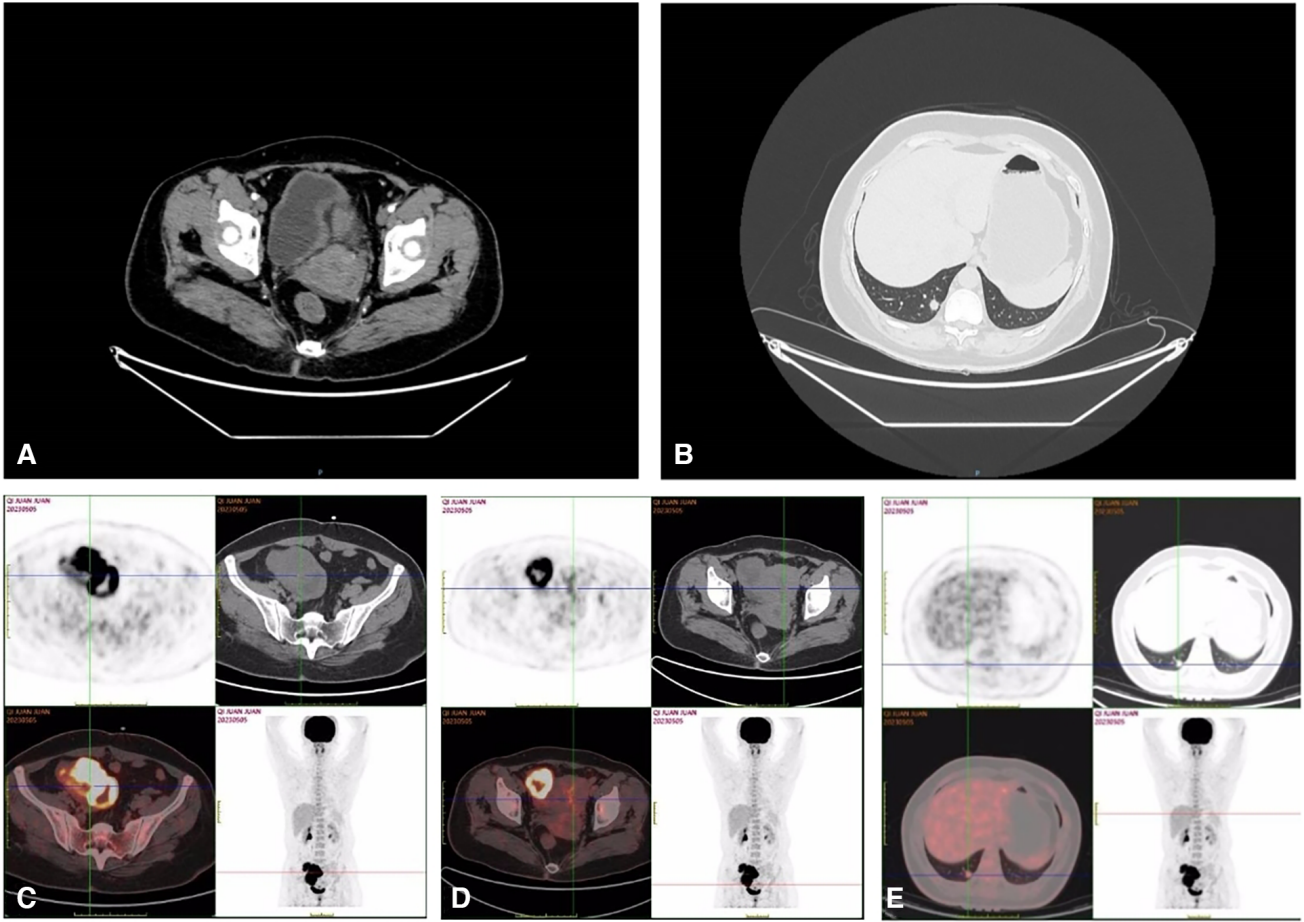
Pelvic CT showed huge mass in the right adnexal area (**A**); chest (lung) CT showed nodule in the lower lobe of the right lung (**B**); ^18^F-FDG PET-CT showed that the right pelvic cystic solid mass and the mass uptake of FDG increased obviously, SUVmax = 22.8 (**C,D**); ^18^F-FDG PET-CT showed that the hypermetabolic nodule in lower lobe of right lung, SUVmax = 3.8 (**E**).

Complementary imaging via Pelvic Computed Tomography (CT) scans, both non-contrast and contrast-enhanced, identified a conglomerate cystic-solid mass of mixed density located in the right pelvic region (Figure 1A). The contrast-enhanced scans illustrated marked heterogeneous enhancement within the solid component, with no observable enhancement within the cystic area. The mass measured approximately 9.0 cm × 6.6 cm. Additionally, vessel tortuosity and thickening were observed in the right adnexal region. These findings are indicative of a complex cystic-solid pelvic mass, likely originating from the right adnexal region, with a high probability of malignancy. The chest Computed Tomography (CT) scans, both non-contrast and contrast-enhanced, revealed the presence of a solid nodule within the subpleural posterior basal segment of the right lower lobe (Figure 1B). This nodule, characterized by spiculated margins and exhibiting signs of pleural retraction, measured approximately 1.1 cm × 0.9 cm. Following the administration of contrast medium, a mild enhancement was observable within the nodule. The constellation of these radiographic features strongly indicates a malignant etiology, thus warranting additional diagnostic evaluations. Subsequently, the Positron Emission Tomography/Computed Tomography (PET/CT) scan reveals a complex cystic-solid mass in the right pelvic region (Figures 1C,D). The mass measures approximately 5.7 cm × 7.8 cm and exhibits a maximum Standardized Uptake Value (SUVmax) of 22.8, a pattern suggesting the strong possibility of ovarian carcinoma. In tandem, the endometrial cavity displays mild widening with elevated radiotracer uptake and an SUVmax of 4.9, which may indicate a physiological state. The scan also identifies a solid nodule in the subpleural posterior basal segment of the right lower lobe (Figure 1E). The nodule measures around 1.0 cm × 1.1 cm with an SUVmax of 3.8, prompting concerns of malignancy and necessitating additional diagnostic efforts to establish whether this represents a primary neoplasm or metastasis. Notably, the lymph nodes in the pelvic cavity and in the hilum and mediastinum show no signs of abnormal radiotracer uptake.

**Figure 2:**
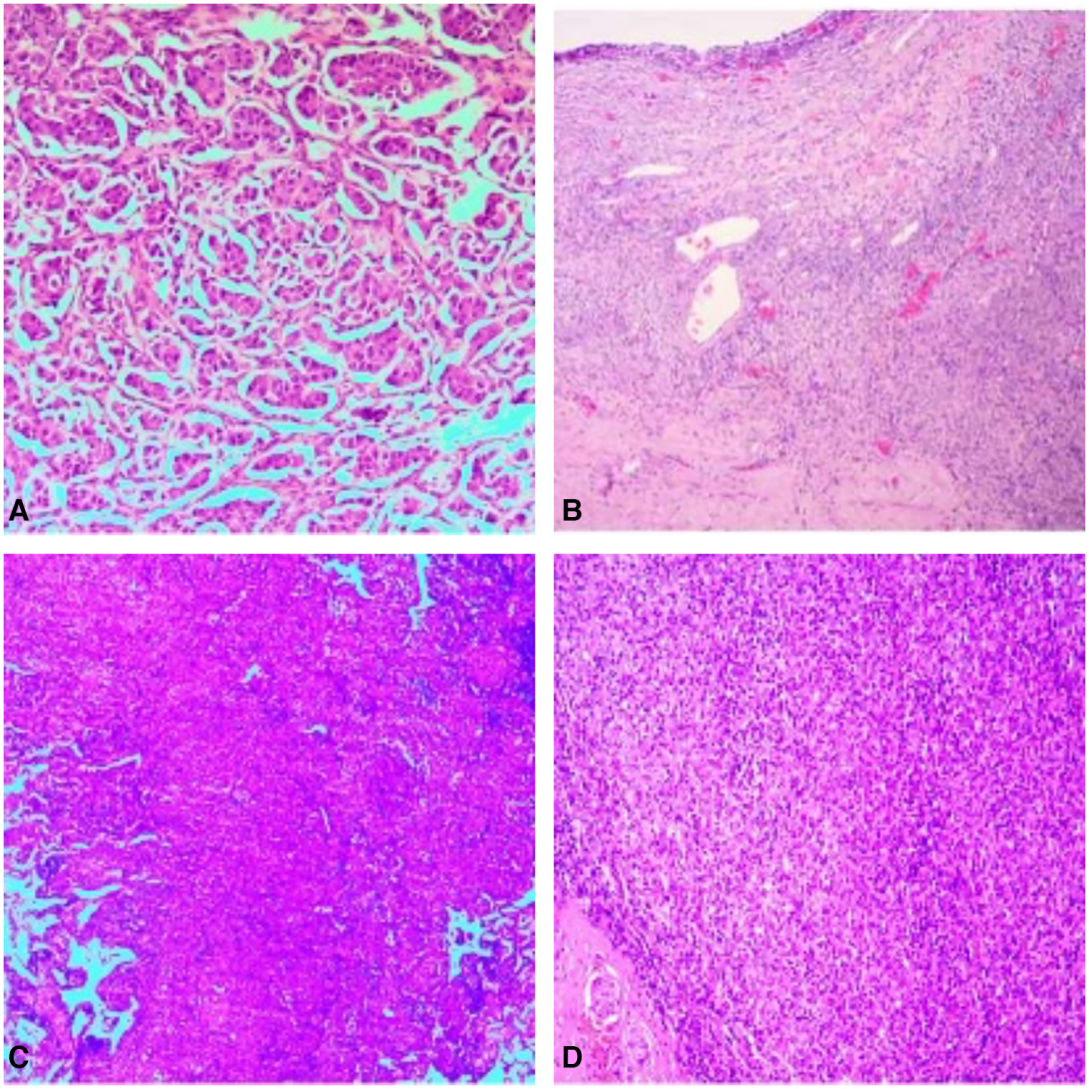
Pathology of the right adnexa showed metastatic adenocarcinoma, mainly solid adenocarcinoma, a few micropapillary carcinoma, pulmonary origin (**A**); pathological picture of uterus and left adnexa, pelvic cavity lymph node, right pelvic cavity lymph node, para-aortic lymph node and part of omentum majus after radical total hysterectomy and double adnexectomy, there was no metastasis (**B**); pathology of the nodule in the lower lobe of the right lung showed invasive adenocarcinoma of the lung, the micropapillary pattern (MPP) accounts for approximately 3% (**C**); pathological picture of examined cancer metastatic lymph nodes (group 7 1/1, group 9 0/1, group 10 0/1) (**D**).

Laboratory analyses revealed significant aberrations in the patient's biomarker profiles. An extraordinarily elevated level of the carbohydrate antigen CA125 was registered at 1,613.00 U/ml, well above the accepted threshold of 0–47 U/ml. Concurrently, the concentration of human epididymal protein 4 displayed a marked elevation, recording at 371.00 pmol/L, which supersedes the normal range of 0–140 pmol/L. Both the postmenopausal and premenopausal Risk of Ovarian Malignancy Algorithm (ROMA) indices manifested substantial elevations, with readings of 96.98% and 92.71% respectively, each exceeding their corresponding reference intervals. The serum carcinoembryonic antigen (CEA) showed a noteworthy escalation, identified at 154.29 ng/ml, which significantly outreaches the typical boundary of 0–5 ng/ml. Intriguingly, while the alpha-fetoprotein (AFP) remained within the normal limit, registering a value below 1.30 ng/ml, the carbohydrate antigen 199 (CA 19-9) demonstrated a remarkable increase, gauged at 1,604.55 U/ml, significantly surpassing the standard reference range of 0–34 U/ml. In essence, the convergence of these deviant laboratory findings strongly intimates the existence of a malignancy. After thorough investigation by a multidisciplinary oncology team, it has been determined that the mass in the right ovary and the nodule in the lower lobe of the right lung are separate primary tumors, with no evidence of metastatic linkage. Based on these conclusions, a two-stage surgical strategy has been proposed: the initial stage will involve a gynecological operation, followed by a thoracic surgical procedure in the subsequent stage. During the operative process, a resection was performed on the right adnexa, with subsequent immediate pathological analysis revealing a malignant tumor. Consequently, the gynecological team proceeded to implement a comprehensive surgical intervention that encompassed total hysterectomy, bilateral adnexectomy, and lymph node dissection. Pathological examination of paraffin-embedded tissue underscored the presence of a poorly differentiated carcinoma. The composite of immunohistochemical markers substantiated the classification as metastatic adenocarcinoma, chiefly TTF-1 and NapsinA positive, solid in nature with trace micropapillary features, pinpointing the lung as the neoplastic source (Figure 2A). And the results of the lung cancer seven serum autoantibodies showed increased levels of CAGE (16.7 U/ml) and GAGE7 (54.0 U/ml), other indicators remained within normal range. Pathology after surgery showed no carcinomatous component in the uterus and the left adnexa, pelvic cavity lymph node, right pelvic cavity lymph node, para-aortic lymph node and part of omentum majus (Figure 2B). Upon the establishment of these diagnostic conclusions, the patient was transitioned to the thoracic surgery department for continued oncological management. A thoracoscopic lobectomy and systematic lymph node dissection were conducted. Paraffin-embedded pathology examination corroborated the manifestation of invasive adenocarcinoma, with salient features including airspace dissemination and extensive affliction of the pulmonary pleura, bronchioles, and nerve structures (Figure 2C). Lymph node pathology presented metastatic infiltration in the seventh group of lymph nodes (Figure 2D). The patient did not receive postoperative chemotherapy due to personal reasons. Molecular pathology gene testing for lung tumor specimens after surgury revealed ALK gene rearrangement (EML4exon6—ALKexon20) positive in this patient (Table 1). The patient received targeted therapy with ensatinib after surgery. At present, the patient is in good condition 3 months after surgery, and no cancer metastasis was found in reexamination.

**Table 1:** Molecular pathological gene detection report of lung tumor tissue.

Content of detection		Sample type	Testing result	Interpretation of results
EGFR Exon18	Gene mutation	Tumor tissue		No mutation
EGFR Exon19	Gene mutation	Tumor tissue		No mutation
EGFR Exon20	Gene mutation	Tumor tissue		No mutation
EGFR Exon21	Gene mutation	Tumor tissue		No mutation
KRAS Exon2	Gene mutation	Tumor tissue		No mutation
KRAS Exon3	Gene mutation	Tumor tissue		No mutation
ALK	Gene rearrangement	Tumor tissue	EML4exon6—ALKexon20	Positive
ROSI	Gene rearrangement	Tumor tissue		Negative

## Discussion

Lung cancer is now the second most diagnosed cancer globally, after breast cancer (4). Metastasis of lung cancer commonly involves the liver, bone, brain, and adrenal glands (5). While lung cancer metastasizing to ovary is exceptionally rare. In a study of lung cancer, ovarian metastases are observed in about 5% of lung cancer patients during post-mortem examinations (6). It appears that the functional status of the ovary renders it more susceptible to metastatic tumors (7). In a study conducted by Karsh, a total of 72 cases of metastatic ovarian tumors were examined from a pool of 10,273 autopsies, with the average patient age being 50.1 years and the highest incidence observed in the fourth decade of life (8). In the current case, the patient was diagnosed with ovarian metastasis at the age of 43. The prevailing viewpoint suggests that the earlier age of onset may be attributed to the ovaries' abundant blood supply and heightened reproductive function during the premenopausal period, which provides a favorable environment for tumor metastasis and growth. Sternberg proposed that the ovarian microenvironment provides a conducive milieu for the growth of metastatic adenocarcinoma (9). Firstly, ovary is situated in the lower region of the pelvis and abdomen, rendering it susceptible to implantation metastasis. Furthermore, inflammatory stimulation and adaptive repair processes that occur in conditions such as annexitis and pelvic inflammatory disease can create a favorable environment for the implantation of cancer cells in the ovaries, thereby, promoting the initiation and progression of cancer.

Diagnosing ovarian metastasis from lung cancer can be challenging, especially when the primary lung cancer histology is adenocarcinoma with the relative rarity of this metastatic pathway in medical literature. Notably, conventional imaging and clinical deductions based on disease prevalence may fail to provide unambiguous insights, especially when confronted with unusual metastatic pathways. In this case the patient presented with lower abdominal pain and no respiratory symptoms. The PET/CT scan showed that the ovarian tumor about the size 5.7 cm × 7.8 cm and the SUVmax is 22.8, the lung nodular, with a burr at the edge, about the size 1.0 cm × 1.1 cm and the SUVmax is 3.8. The PET/CT tomography showed no obvious abnormal metabolism in the rest. The volume of ovarian tumor was significantly larger than that of lung tumor in this patient. The levels of CA125 and ROMA indices was significantly elevated. And based on the patient's radiological interpretations and biomarker profiling, the patient was initially misdiagnosed as double primary tumor in the ovary and lung. Ovarian metastases from lung cancer are uncommon, but clinicians should take into consider the possibility especially in patients with lung mass. There is no confident diagnostic tool for the differential diagnosis between primary and metastatic lung cancer, and surgery is frequently needed to make the diagnosis as well as for symptom relief in most cases. Immunohistochemical staining of postoperative specimens is an important adjunctive component in the evaluation of a neoplasm, whether primary or metastatic, with important help to treatment decision making. It has been shown that specific markers such as TTF-1 and Napsin A play a crucial role in identifying lung adenocarcinoma in immunohistochemical (10). This case further reinforces the necessity of employing immunohistochemistry, particularly in scenarios where traditional morphologic assessments may not conclusively establish a diagnosis. The case also accentuates the importance of a multidisciplinary team approach in managing complex and unanticipated cancer presentations, as evidenced by the crucial cooperation between the gynecology and thoracic surgery departments in this case. The revelation of the ovarian neoplasm's metastatic origin resulted in an essential shift in management to thoracic surgery, underscoring the importance of adopting an adaptable treatment approach responsive to evolving diagnostic evidence. This adaptability has profound implications for altering patient prognosis and survival rates. In the lung tumor specimens after thoracic surgery, a micropapillary pattern (MPP) was identified, accounting for approximately 3% of the lesions observed (Figure 2C). The MPP is characterized by the presence of micropapillary structures without a fibrovascular core (11). Previous studies have indicated that the MPP-positive cases tend to exhibit higher rates of poor prognostic factors such as visceral pleural invasion compared to MPP-negative cases in early-stage tumors (11). It is significantly higher in female patients, particularly nonsmokers (11). The presence of MPP might indicate the possibility of a poorer prognosis for early-stage lung adenocarcinoma and alert clinicians to conduct more thorough searches for metastases. Furthermore, the detection of seven specific lung cancer tumor-associated serum autoantibodies has diagnostic value in detecting early-stage lung cancer (12). It includes p53, CAGE, PGP9.5, GBU4-5, SOX2, MAGE A1, and GAGE7.The combined detection of seven serum autoantibodies has been identified as an indicator for early screening and diagnosis of lung cancer, compensating for the low sensitivity of traditional tumor markers in early-stage lung cancer. It has been demonstrated that the clinical utility of seven serum autoantibodies achieved a sensitivity of 67.5% and a specificity of 89.6% for the early detection of lung cancer in a previous study (13). In this specific case, the results of the lung cancer seven serum autoantibodies showed increased levels of CAGE (16.7 U/ml) and GAGE7 (54.0 U/ml), while other indicators remained within normal range.

At present, immune-targeted drugs play an important role in the treatment of malignant tumors. Lung molecular pathology gene testing revealed ALK gene rearrangement (EML4exon6—ALKexon20) positive in this patient. ALK gene rearrangements are detected in approximately 3%–7% of non-small cell lung cancer (NSCLC) patients (14). The fusion gene EML4-ALK involves echinoderm microtubule-associated protein-like 4 (EML4) and anaplastic lymphoma kinase (ALK) and is recognized as one of the most critical pathogenic driver genes in NSCLC (15). Currently, the incidence of ovarian metastasis in NSCLC patients with ALK rearrangement-positive is uncertain. Some studies suggest that ALK gene rearrangement may increase the risk of ovarian metastasis in NSCLC patients (16). Physicians should be aware of the possibility of ovarian metastasis in female LUAD patients with ALK rearrangement-positive. The incidence of ovarian metastasis in female patients with ALK rearranged LUAD needs to be confirmed by further studies (17). Previous studies have demonstrated ALK inhibitors have significant benefits in terms of prolonging progression-free survival and overall survival in patients with ALK-positive advanced NSCLC when compared to chemotherapy (18). Currently, routine gene mutation testing for the EML4-ALK fusion gene is performed for NSCLC patients in clinical settings. Therefore, targeted therapy with ensartinib was administered in this patient. Due to the relatively short follow-up period after the patient's discharge, we were unable to assess the long-term prognosis of this patient with ovarian metastases from lung cancer treated with surgery combined with targeted drug therapy.

## Conclusion

Indeed, small nodular lung adenocarcinoma with ovarian metastasis is considered rare. The accurate identification of primary tumors relies on postoperative pathology and immunohistochemical analysis. The unique trajectory of patient management in this case offers valuable insights into the treatment strategies necessitated by such uncommon occurrences. In summary, this case sheds light on the diagnostic and treatment complexities associated with a rare metastatic pattern of lung cancer, stressing the continued need for research, vigilance, and a flexible, multidisciplinary approach in oncology.
